# The assessment method of foreign language communication ability of intelligent emotional network based on artificial emotion

**DOI:** 10.3389/fpsyg.2022.975359

**Published:** 2022-09-20

**Authors:** Chen Li

**Affiliations:** School of Foreign Languages, Huanghuai University, Zhumadian, Henan, China

**Keywords:** artificial emotion, intelligent emotion network, foreign language communication ability, assessment method, emotions

## Abstract

The traditional evaluation methods of foreign language communication skills cannot deal with emotional information in the process of communication. Psychologists believe that a real personalized evaluation system should be smart. Based on the emotion network technology of artificial emotion intelligence, aiming at the shortcomings of the traditional evaluation system, this paper puts forward a new language ability evaluation system with certain emotion judgment function. The system can easily obtain and identify emotions in foreign communication, and can also carry out individual learning at the cognitive and emotional levels on the basis of comprehensive analysis of emotions and communication effects.

## Introduction

Foreign language communication is the complete process of receiving signal stimuli, generating a series of responses and expressing them correctly, which is a combination of body functions and body structures (Kong, 2020). Artificial emotion (AE) is a new research direction in the field of intelligent control that has received increasing attention in recent years. With the increasing maturity and sophistication of industrial robotics, robots are gradually entering medical, health care, home, and sports (e.g., robot soccer), which are not satisfied in the current intelligent control theory, and therefore, there is an urgent need to find new approaches (Li et al., 2020). Reaction, a relatively low level biological response such as the classical reflex mechanism in animals, has been more attempted in the study of coordinated control of robots and autonomous robots, but only for simple tasks (He and Sun, 2021). Therefore, the evaluation of foreign language communication skills is essentially a comprehensive evaluation of several indicators.

Emotional process, mainly including emotions and emotions, is the experience and accompanying psychosomatic changes produced in the heart of human beings toward the objective world (Cao et al., 2021). An intelligent emotional network of artificial emotions is a functional approximation of these two aspects of emotional processes in order to improve the intelligence and autonomy of intelligent systems. Palanisamy et al. (2021) proposing that emotions can provide the autonomy required by robots and are the ultimate source of intelligence (Alsubari et al., 2022). Even argued that without emotions, robots cannot exhibit intelligent behavior.

As for the assessment of foreign language communication ability, there are many problems, and the use of related techniques such as sentiment analysis can solve such problems (Radwan et al., 2017). Specifically, it can be divided into 3 areas of research: sentiment analysis based on sentiment lexicon (features). To solve this problem core is to build sentiment lexicon (Yingjie et al., 2017). At present, there are many commonly used sentiment dictionaries, such as HowNet, a dictionary from Zhiwang, NTUSD from Taiwan University, Li Jun Chinese positive and negative dictionary from Tsinghua University (Yinxiang, 2017). Sentence-level distributed representation algorithms based on deep learning can obtain sentence or paragraph distributed representation by compounding from word distributed representation, thus enabling the application of sentence or paragraph distributed representation to text sentiment analysis tasks (Huang S.^2021^).

## Related work

The modeling of intelligent emotional networks for artificial emotions is broadly divided into two categories, namely, traditional analytic and symbolic (analytic and symbolic) models and synthetic bottom-up (synthetic bottom-up) models.

**FIGURE 1:**
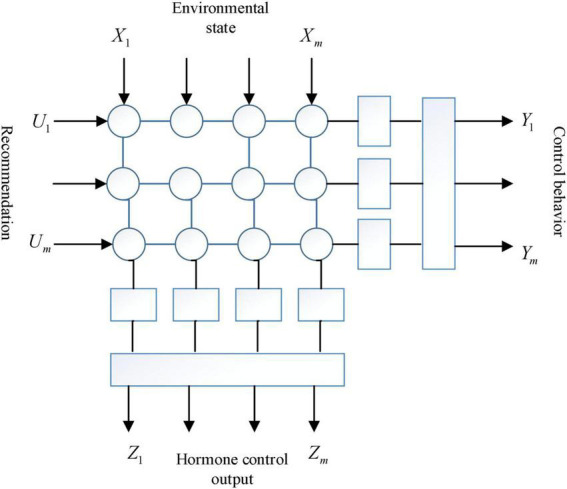
Cross-array module.

This is the more traditional modeling idea that strives to be consistent with the mainstream of psychological research and tries to embody all the various features of human emotions in the model, so it tends to be more complex (Yu and Peng, 2021). Based on this view, (Wan and Tang, 2021) slacken the abstraction of emotions into 22 most basic emotions and categorize them separately in the above mentioned evaluation categories. Yi and Mandal (2019) proposing a model of an intelligent body with emotions that contains deep factors of the emotion generation process; the hormonal system generates emotions, moods and other physical or mental states; and the genetic system influences the other two major systems through genes and genetic factors, of which the crossbar array-based (crossbar array) module is shown in Figure 1. Ali et al. (2017) proposing a computational model with a four-layer structure containing multiple functional modules such as the Response and Emotion Engine. These are models of qualitative and semantic symbolic representations of emotions that contain sufficient complexity from a psychological perspective. A review of such models is presented by Liu Q. et al. (2019). However, Huang (2018) argues that these models are Overdesigned and overly complex, and thus not applicable to intelligent control. In summary it is very feasible to build an intelligent emotional network for artificial emotions.

The array module is divided into single-mode array module and multi-mode optical module according to the applicable fiber types. The fiber wavelength of the single-mode array module is 1310 ηm. 1550 nm and WDM wavelength, while the fiber wavelength of multimode array module is 85 σ Nm or 131 nm, mainly 85 nm optical fiber wavelength at present.

**FIGURE 2:**
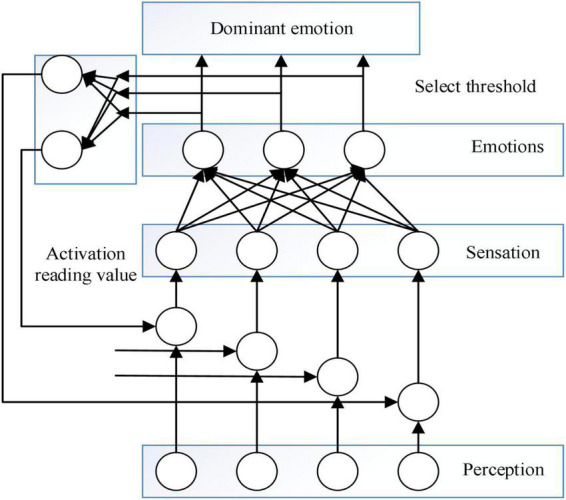
Emotional model.

In order to be able to implement intelligent emotion networks of artificial emotions in concrete intelligent systems, it is necessary to reduce the complexity of emotion models, and this new approach to emotion modeling emerged. In Huang D. (2021), a relatively simple emotion model as shown in Figure 2 was designed based on a specific task and environment, using a bottom-up analysis approach to move from the abstract to the concrete and to capture the main factors that affect the system performance. The model considers sensation, perception, emotion, and hormone systems, and emotion exhibits the following four characteristics:

1) Emotion is measured numerically, i.e., it can be expressed as a positive or negative number.
2) A certain emotional state lasts for a certain period of time, and sudden changes between different emotions are not allowed, especially for two very different emotions;
3) The generation of a certain emotion is not only dependent on the current feeling but also related to its recent emotional state.
4) The current emotional state can influence or even distort the subject’s perception.

The model contains four basic emotions, namely: happiness, sadness, anger, and fear, all closely related to the state of the subject and the environment in which he or she lives. Dimitrievska and Ackovska (2020) pointed out that there are two problems with pre-emotional modeling (e.g., OCC):

1) Emotions evaluate something as an absolute good/bad concept that changes too quickly.
2) When emotions evaluate the degree to which something satisfies multiple goals, they usually consider the degree to which each goal is satisfied separately, and lack integration.

Therefore, they proposing a fuzzy model of emotion based on fuzzy goal satisfaction and influence level, and fuzzy mapping of events to goals. The structure is the same as Gadanho’s model, but fuzzy reasoning is used for the two aspects of external stimuli leading to emotion generation and emotional states leading to behavior, solving the two previously mentioned problems. Alemi (2016) also used fuzzy logic to build a model based on perception (feeling), emotion (mood) and affect, arguing that a mathematical model can be used to represent the interactions between the three. Iskenderhakki (2015) proposing a computational model of emotion from the perspective of intelligent speech signal processing, whose basic structure follows the mainstream theory of psychology from sensory stimulus to emotion to behavior (Yang et al., 2014; Liu et al., 2016; Liu Z. et al., 2019).

The influence of English application environment English communication is the embodiment of English application ability based on listening and speaking. It is mainly manifested in the capture, input, analysis and output of information in English. If we have a good environment for information input, we can obtain English information, think and analyze, then there are many opportunities to output in English. Input and output are proportional. Generally, there is no such a complete input environment of English language around us. Even if there are, many of them are deliberately created artificial environments, which have many disadvantages and flaws. So the first factor that affects our English communication is environmental factors. We need a communicative environment with a strong flavor of English.

Although the above models are very imperfect with respect to the rich emotional life of humans, their simple structure allows them to be applied in various intelligent systems.

## Methodology

The video interface in the interaction model can transmit the image of the subject obtained from the video input device at the subject’s side to the emotion model, and the emotion assessment model formed by the emotion model is displayed on the subject’s side. The video interface in the interaction model transmits the subject’s image obtained from the video input device at the subject’s side to the emotion model and displays the emotion guidance information formed by the emotion assessment model at the subject’s side.

**FIGURE 3:**
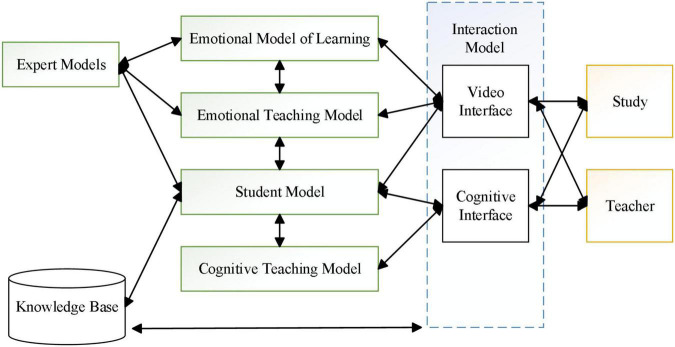
Functional knot of the intelligent emotion network assessment system.

**FIGURE 4:**
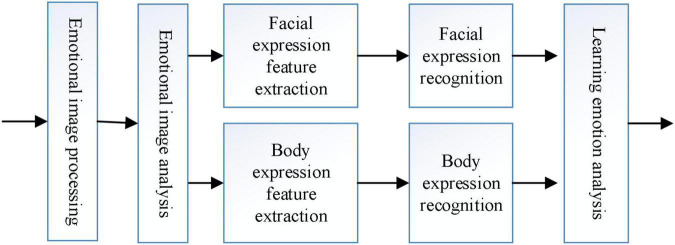
Structure of the emotion model.

As in Figures 3, 4 the emotion model firstly preprocesses the acquired subject’s emotion image, then extracts the subject’s facial expression features and body expression features on this basis, and recognizes the subject’s facial expressions and body expressions according to the corresponding emotion model, and finally performs a comprehensive analysis of the subject’s expressions to obtain the subject’s emotion.

**FIGURE 5:**
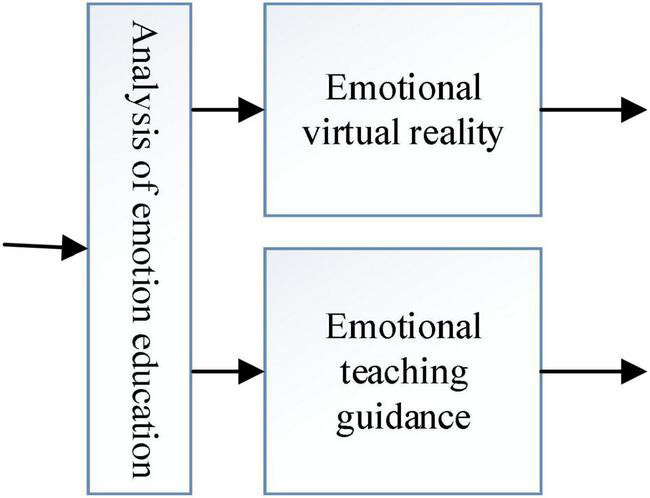
Functional structure of the emotion assessment model.

As in Figure 5 the emotion assessment model is mainly based on the emotion of the subject obtained from the emotion model and the cognitive characteristics of the subject in the subject model, and carries out emotion assessment analysis according to the emotion assessment principle of educational psychology, and provides personalized emotion assessment guidance for the subject, and uses virtual reality technology to realize the visualized emotional communication between the assessment system and the subject.

**FIGURE 6:**
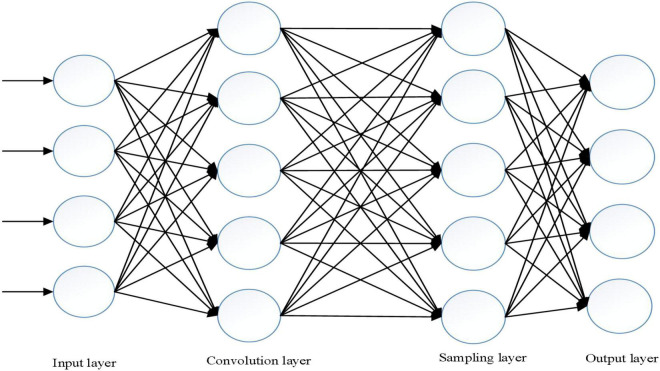
Basic model diagram of intelligent emotional network.

When the objective function is obtained, the network model feeds the error forward from the last layer to the last layer by calculating the error through a back propagation algorithm. The sub-process is called the backward feedback operation. The basic model of intelligent sentiment network is shown in Figure 6.

**FIGURE 7:**
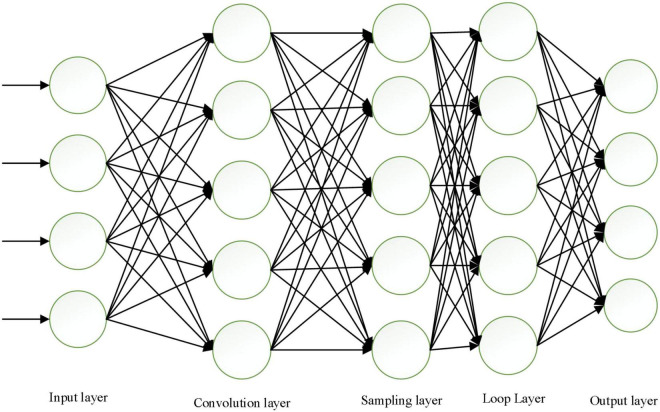
Improved model diagram of intelligent emotional network.

The improved intelligent sentiment network model is shown in Figure 7.

The specific training process is: (1) the network is initialized with the weights; (2) the input layer is fed with the original data; (3) the implicit layer performs convolutional operations, sampling, and forward propagation from the fully connected layer to the higher network layers; (4) the cyclic implicit layer calculates the error by cycling; (5) if the calculated error is greater than the target expectation, the error is back-propagated back to the network model, and the errors of the fully connected layer and sampling layer and the error of the sentiment calculation layer. If the final error is less than or equal to the desired error value, the training is finished; (6) The weights are updated according to the average value of the errors obtained in the three cycles.

**FIGURE 8:**
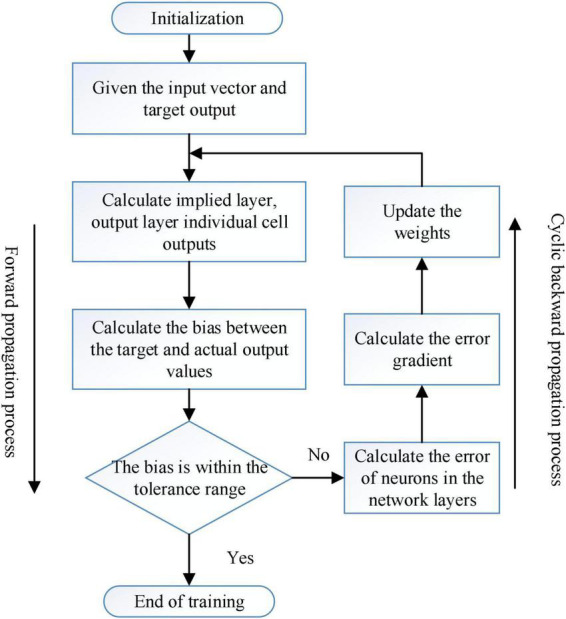
Improved training flowchart of convolution neural network.

The improved intelligent sentiment network training process is shown in Figure 8.

(1) Forward propagation process of the sentiment computation layer

The convolution operation of the sentiment computation layer of the network model is called the forward propagation process of the sentiment computation layer. The sentiment computation layer uses a step-specific convolution operation to obtain the initial operator function and perform a convolution operation with that accident operator function (for semantic sentiment recognition, i.e., weighted sum plus a bias for the eigenvalues of the weight matrix and text sentiment), and then the output of the layer is obtained by the activation function.

(2) Forward propagation process of the sampling layer

The feature data obtained from the sentiment calculation layer is transferred to the sampling layer as input, and the input data is downsampled by the pooling operation in the downsampling layer, so as to avoid the overfitting situation. The maximization pooling method is the process of picking up the semantic sentiment peaks. The mean pooling method is the process of picking up the semantic sentiment mean. The random pooling method picks up the probability of all sentiment features appearing in all features, and then randomly selects one of them as the semantic sentiment feature value, in which the larger the probability is, the higher the probability of the feature being selected in the end.

(3) Forward propagation process of the fully connected layer

After obtaining the sentiment features from the sentiment calculation layer and sampling layer, the extracted sentiment features are input to the fully connected layer in the model. And the extracted sentiment features are classified to obtain the classification model and get the output matrix. Then it is passed into the next layer after activation by the excitation function *f*(*y*).

**FIGURE 9:**
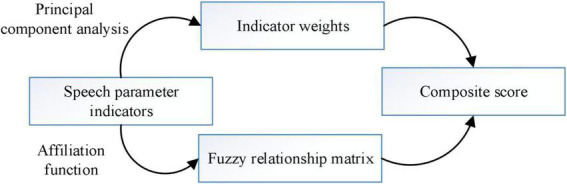
Fuzzy integrated assessment framework diagram.

A fuzzy comprehensive assessment model based on affective components is further constructed. In view of the complexity of the human body system, the multi-source nature of factors affecting foreign language communication, and the fuzziness of the influencing factors, this paper adopts fuzzy comprehensive evaluation for multivariate information fusion of multiple language indicators to quantitatively assess the foreign language communication ability level. Affective component analysis is used to determine the multiple indicators of the influencing factors and calculate their weights separately. The fuzzy values and uncertainties are dealt with by the fuzzy comprehensive assessment method. The key techniques to be solved by the fuzzy comprehensive assessment method include determining the weights of the indicators and selecting the affiliation function. The evaluation process is shown in Figure 9.

The final assessment results are directly related to the selection of weights. If the weight of a parameter is overemphasized, the importance of other items is bound to be weakened; if there is a serious deviation from the normal value of weights, the assessment model is not usable. There are many methods to determine the weights of index parameters, and the sentiment component analysis method, which determines index weights from objective data, is a quantitative method to determine index weights with a certain degree of objectivity and scientificity.


(1) $$\left\{ \begin{array}{c} F_{1}=a_{1,1}\,x_{1}+a_{2,1}\,x_{2}+\ldots +a_{p,1}\,x_{p}\\ F_{2}=a_{1,2}\,x_{1}+a_{2,2}\,x_{2}+\ldots +a_{p,2}\,x_{p}\\ \ldots \,\ldots \\ F_{m}=a_{1,m}\,x_{1}+a_{2,m}\,x_{2}+\ldots +a_{p,m}\,x_{p} \end{array}\right.$$

The sentiment components are *F*_1_, …, *F*_*m*_ a total of *m*, *a*_1,1_, …, *a*_*p*,*m*_ the eigenvectors corresponding to the eigenvalues λ_1_, …, λ_*m*_ of the covariance matrix *con*(*X*) of the original dataset *X* = (*x*_1_, *x*_2_, …*x*_*p*_) and *x*_1_, *x*_2_, …*x*_*p*_ the vectors after normalization of the dataset.

The contribution rate of sentiment components is calculated according to Equation (2).


(2) $$c_{i}=\frac{\sum_{k=1}^{i}\lambda_{k}}{\sum_{k=1}^{m}\lambda_{k}}\left(i=1,\ldots ,m\right)$$

For the sake of narrative convenience, let the first and second sentiment components *F*_1_,*F*_2_ correspond to a contribution rate of *c*_1_,*c*_2_, whose sum is *c*_1_ + *c*_2_ > 85%, then the first two sentiment components are extracted to describe the original information.

Based on the sentiment component analysis method to find the weight of linguistic indicators is as in Equation (3).


(3) $$\begin{array}{c} w_{1}=\frac{a_{1,1}\times c_{1}+a_{1,2}\times c_{2}}{c_{1}+c_{2}}\\ \ldots \\ w_{p}=\frac{a_{p,1}\times c_{1}+a_{p,2}\times c_{2}}{c_{1}+c_{2}} \end{array}$$

The factors affecting foreign language communication include simple or complex comprehension, judgment, expression, repetition, naming, memory and attention to things seen or heard or instructions, which are summarized into nine variables, namely *U* = {auditory ability, visual ability, attention, memory, comprehension, judgment, expression, average energy of speech, average speed of speech}. The set of ability level rubrics *V* = {intact, mildly dysfunctional, moderately dysfunctional, severely dysfunctional}, and the fuzzy values of ability levels were set as *V* = {0.90, 0.75, 0.55, 0.25}.

The factors affecting the foreign language communication level are difficult to be described by a precise mathematical model, so a fuzzy mathematical method is used to estimate the factor ability level values. A certain influencing factor is calculated as in Equation (4).


(4) $$r=\frac{0.5}{H-M}\times \left(c\,u\,r\,\_\,v\,a\,l-M\right)+0.5$$

For computational convenience, the ability level fuzzy value as the segmentation function cut-off point, and *k* is set to 1.2. *v*_1_ ∼ *v*_4_ The corresponding fuzzy membership functions are shown in Equations (5) to (8).

*v*_*1*_ Fuzzy membership functions


(5) $$F_{1}\,\left(x\right)=\left\{ \begin{array}{cc} 0, & x<0.75\\ {\left(\frac{x-0.75}{0.9-0.75}\right)}^{1.2}, & 0.75\leq x\leq 0.9\\ 1, & x>0.9 \end{array}\right.$$

*v*_*2*_ Fuzzy member functions


(6) $$F_{2}\,\left(x\right)=\left\{ \begin{array}{cc} 0, & x<0.55\\ {\left(\frac{x-0.55}{0.725-0.55}\right)}^{1.2}, & 0.55\leq x\leq 0.725\\ 1, & 0.725\leq x\leq 0.775 \end{array}\right.$$

*v*_*3*_ Fuzzy member functions


(7) $$F_{3}\,\left(x\right)=\left\{ \begin{array}{cc} 0, & x<0.25\\ {\left(\frac{x-0.25}{0.455-0.25}\right)}^{1.2}, & 0.25\leq x\leq 0.455\\ 1, & 0.455\leq x\leq 0.545\\ {\left(\frac{0.75-x}{0.75-0.545}\right)}^{1.2}, & 0.545\leq x\leq 0.75\\ 0, & x>0.75 \end{array}\right.$$

*v*_*4*_ Fuzzy member functions


(8) $$F_{4}\,\left(x\right)=\left\{ \begin{array}{cc} 1, & x<0.25\\ {\left(\frac{0.55-x}{0.55-0.25}\right)}^{1.2}, & 0.25\leq x\leq 0.55\\ 0, & x>0.55 \end{array}\right.$$

Since the maximum affiliation principle method loses too much information, the results obtained too reluctantly may be wrong. In this paper, we use the weighted average principle to find the affiliation level, and the affiliation degree obtained from each level is used as the weight to normalize the weighted sum of all the level values, and the final composite score u*is obtained by adopting the principle of the closest neighboring value for the foreign language communication level.


(9) $$u^{*}=\frac{\sum_{i=1}^{n}V\,\left(v_{i}\right)\cdot b_{i}}{\sum_{i=1}^{n}b_{i}}$$

## Experiments

First we assessed the emotions during the communication process, and the specific data distribution is in Tables 1, 2.

**TABLE 1:** Distribution of semantic emotional labels.

Emotional label	Total number of samples
Satisfaction	3228
Disappointment	1858
Admiration	2195
Reproach	7459
Like	9500
Dislike	16539

**TABLE 2:** Distribution of auxiliary emotional labels.

Auxiliary tag	Total number of samples
Reviews for purchase experience	4088
Views for service providers	9225
Reviews for goods	28175

In order to illustrate the performance of our intelligent emotional network in judging foreign language ability, we compared it with DL method and ML method. The specific comparison results are shown in Tables 3, 4. It can be seen that our method has very significant advantages in language ability evaluation before and after improvement.

**TABLE 3:** Comparison of language communication ability evaluation results.

Models	Loss 1	Loss 2	Error 1	Error 2	Accuracy
ML-based	0.2915	0.0915	0.4865	1.8514	0.4666
DL-based	0.2893	0.0907	0.4858	1.8435	0.4675
Ours	0.1372	0.0843	0.3149	1.8140	0.7917

**TABLE 4:** Comparison of improved language communication ability evaluation results.

Models	Loss 1	Loss 2	Error 1	Error 2	Accuracy
ML-based	0.2895	0.0909	0.4860	1.8437	0.4677
DL-based	0.1374	0.0845	0.3151	1.8142	0.7919
Ours-improved	0.1292	0.0522	0.2344	1.6124	0.8127

As can be seen in Figure 4, the impact of different batches on the neural network model was evaluated in 20 steps from a data size of 10–100.

**FIGURE 10:**
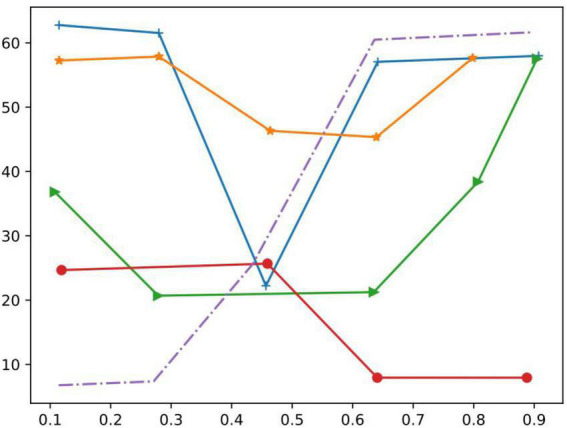
Influence of different batch size and epochs on emotional network.

In this paper, the effect of different batchsize on the neural network model is evaluated from a data size of 10–100 in 20 steps, and the results are shown in Figure 10. Therefore, in this paper, the batchsize parameter is adjusted to 100 and the epochs parameter is adjusted to 20.

We compared it with DL method and ML method. Evaluating the effect of different batches on the neural network model in 20 steps from a data size of 10–100 shows that our method has a very significant advantage in the evaluation of language ability before and after improvement.

**FIGURE 11:**
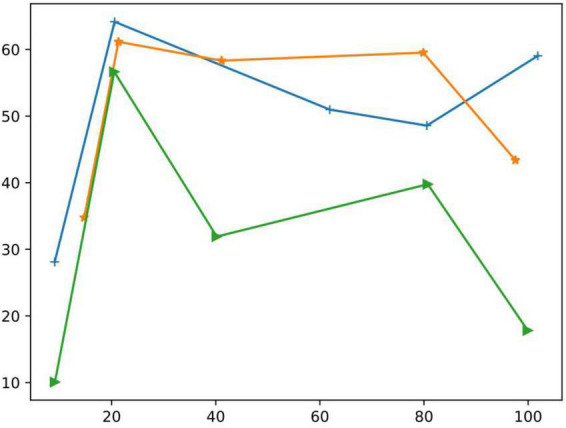
The influence of learning rate and momentum factor on the model.

The momentum coefficient determines the influence of the weight of the previous batch on the weight of the current batch. In order to select the optimal value, we study the influence of different optimal values on the neural network, as shown in Figure 11 (The X-axis is the epochs parameter and the Y-axis indicates the general parameter).

We evaluate the communication ability, due to the large space occupied by data calculation, this paper lists 6 cases of specific data to demonstrate the process of applying the model, as shown in Table 5. Where the first 7 indicators are all percentage data, and labels 1, 2, 3, and 4 indicate intact, mildly dysfunctional, moderately dysfunctional, and severely dysfunctional abilities, respectively.

**TABLE 5:** Data table of foreign language communication ability indicators (%).

Sample sequence	Auditory ability	Visual ability	Memory	Comprehension	Judgment	Expression	Attention	Speech energy (dB)	Speech speed (min)	Label
1	61.41	87.65	29.16	77.79	89.99	54.36	63.99	63.82	54.81	3
2	98.79	92.60	64.59	88.87	98.35	86.21	86.23	61.13	38.32	2
3	68.15	75.91	40.02	83.42	88.89	69.02	67.01	56.00	39.55	2
4	96.31	93.71	76.45	98.91	98.62	94.34	91.32	67.22	40.62	1
5	96.75	94.62	64.59	97.58	78.92	74.52	88.95	74.22	55.86	1
6	23.76	66.69	11.52	6.68	36.68	16.55	27.20	65.31	13.45	4

The data were processed by software and 2 principal components and factor loading moments were obtained array. See Table 6.

**TABLE 6:** Factor loading matrix.

Variable	Factor 1	Factor 2
Auditory ability	0.979	0.018
Visual ability	0.947	0.278
Memory	0.936	0.082
Comprehension	0.975	–0.132
Judgment	0.889	–0.418
Expression	0.959	–0.200
Attention	0.996	0.014
Average energy of speech	0.233	0.972
Average speech speed	0.770	0.130

Both factor analysis and principal component analysis are factor analysis methods, both based on statistical analysis methods, but there are major differences between them: principal component analysis is to extract principal components through coordinate transformation, that is, to transform a set of variables with correlation into a set of independent variables, and to represent principal components as linear combinations of the original observed variables; while factor analysis is to construct factor models, decompose the original observed variables into linear combinations of factors.

Since the coefficients of the factor scoring matrix derived from the SPSS run factor analysis cannot be directly used for the coefficients of the scoring matrix of the principal component analysis, they need to be obtained separately using the principal component scoring formula. The principal component scoring matrix is the principal component model coefficients, and the first two parameters with a cumulative contribution of >85% and an eigenvalue >1 are taken as the principal components. principal components, instead of the original nine parameters with certain correlation, the formula of the principal component model is as follows.


(10) $$\begin{array}{c} F_{1}\,\left(x\right)=0.3703\,x_{1}+0.3547\,x_{2}+0.3502\,x_{3}+0.3684\,x_{4}\\ +0.3358\,x_{5}+0.3627\,x_{6}+0.3767\,x_{7}+0.0878\,x_{8}+0.2911\,x_{9} \end{array}$$

(11) $$\begin{array}{c} F_{2}\,\left(x\right)=0.0151\,x_{1}+0.2457\,x_{2}+0.0718\,x_{3}-0.1162\,x_{4}-\\ 0.3699\,x_{5}-0.1765\,x_{6}+0.0115\,x_{7}+0.8604\,x_{8}+0.1144\,x_{9} \end{array}$$

where, *x*_1_, …, *x*_9_ is the original indicator variable after standardization. From the above formula and according to the method of principal component analysis to find the weights of each parameter, the weight vector *A* is obtained.


(12) A={0.1237,0.1324,0.1204,0.1151,0.0890,0.1096,0.1255,0.0810,0.1034}

Sample 1 is evaluated according to the model proposing in this paper, and the fuzzy value is obtained from Equation (9), and then the fuzzy relationship matrix of sample number 1 is obtained based on the affiliation function of the fuzzy rubric set


(13) $$R_{1}=\left[\begin{array}{cccc} 0 & 0.2979 & 0.6122 & 0\\ 0.8143 & 0.1353 & 0 & 0\\ 0 & 0 & 0.1471 & 0.8364\\ 0.1323 & 0.9732 & 0 & 0\\ 0.9984 & 0.0004 & 0 & 0\\ 0 & 0 & 1 & 0.0104\\ 0 & 0.4496 & 0.4743 & 0\\ 0 & 0 & 0.6874 & 0\\ 0 & 0.2012 & 0.7094 & 0 \end{array}\right]$$

Fuzzy integrated evaluation by weight vector and fuzzy relationship matrix to obtain fuzzy evaluation value


(14) $$B_{1}=A^{{}^{\circ}}\,R=\left(0.2119,0.2440,0.3916,0.1371\right)$$

The affiliation level is calculated by the weighted average principle


(15) $$\begin{array}{c} u^{*}=(0.2119\times 0.9+0.2440\times 0.75+0.3916\times 0.55+0.1371\times \\ 0.25)/(0.2119+0.2440+0.3916+0.1371)=0.633 \end{array}$$

Based on the principle of the closest neighboring value, 0.6331 is in the vicinity of moderate (0.55) in the assessment rubric set, so the subject’s foreign language communication ability is moderate, which is consistent with the actual situation of the case3 (moderate).

The comparison of model assessment and physician assessment results for all study subjects is shown in Table 7.

**TABLE 7:** Comparison of assessment results for different ability levels (*n* = 50).

Assessment method	Competence intact	Mild incapacity	Moderate incapacity	Severe disability
Clinical	22	15	9	4
Model	24	13	10	3

The accuracy of model assessment is high, and the accuracy rates of all four classifications are 80% or above, which basically meet the requirements. In the same way, the affiliation levels u* of samples 2 to 6 were calculated as 0.747, 0.623, 0.830, 0.816, and 0.343, respectively, and were adjacent to 0.75 (mild), 0.55 (moderate), 0.9 (intact), 0.75 (mild), and 0.25 (severe), respectively, in the rubric set, which were consistent with the known results except for sample 3. According to the comprehensive assessment model proposing in this paper, it can be used for quantitative assessment of language proficiency and can give measures of different abilities with reference to the grade assessment results.

## Conclusion

Artificial emotion is a research direction that attempts to simulate the human emotional process in order to obtain intelligence and autonomy that cannot be achieved by rational thinking. Moreover, the random pooling method selects the probability of all emotional features appearing in all features, and then randomly selects one of them as the semantic emotional feature value. The greater the probability, the higher the probability of the final feature selection. Therefore, we propose an artificial emotion-based intelligent emotion network to effectively realize the evaluation of foreign language communication ability. Experimental results show that our designed intelligent emotion network has very significant evaluation performance.

## Data availability statement

The original contributions presented in this study are included in the article/supplementary material, further inquiries can be directed to the corresponding author.

## Author contributions

CL: writing—original draft preparation, article writing, data sorting, and the completion of the whole article.
